# Mediating Role of Intimate Partner Violence Between Emotional Dependence and Addictive Behaviours in Adolescents

**DOI:** 10.3389/fpsyg.2022.873247

**Published:** 2022-05-09

**Authors:** Patricia Macía, Ana Estevez, Iciar Iruarrizaga, Leticia Olave, Mᵃ Dolores Chávez, Janire Momeñe

**Affiliations:** ^1^Faculty of Psychology, University of the Basque Country, San Sebastian, Spain; ^2^Department of Personality, Evaluation and Psychological Treatment, University of Deusto, Bilbao, Spain; ^3^Department of Experimental Psychology, Cognitive Processes and Speech Therapy, Complutense University of Madrid, Madrid, Spain; ^4^Department of Social Work, University Técnica of Manabí, Manabí, Ecuador

**Keywords:** partner violence, emotional dependence, adolescence, violent relationships, addictions

## Abstract

**Objective:**

Intimate partner violence (IPV) has been related to emotional dependence and addictive disorders. This study aims to provide a global approach to analyse the relationship between these variables and to determine the factors underlying permanence in violent relationships.

**Methods:**

It is a non-experimental, cross-sectional correlational design study. Participants had to have at least one dating relationship for at least 1 month to complete the questionnaire, which included the following instruments: emotional dependence scale (DEN), scale of violence in dating relationships (VREP) and impulse control disorders scale (MULTICAGE CAD 4).

**Results:**

The sample consisted of 1.533 adolescents, 53.9% were male (*n* = 826) and 46.1% female (*n* = 707), between 14 and 18 years. Emotional dependence correlated significantly with received violence (*r* = 0.37, *p* < 0.001). Compulsive spending is strongly associated with emotional dependence (*r* = 0.21, *p* < 0.001), whereas sex addiction is associated with received violence to a greater extent (*r* = 0.18, *p* < 0.001). Received violence mediates on emotional dependence and addictions.

**Conclusion:**

IPV is a risk factor for the perpetuation of addictive behaviours. It is advisable to promote affective education for developing resilience and adequate coping.

## Introduction

The existence of addictive behaviours in the adolescence period has been related to emotional dependence. This is defined as an extreme need for affection from the other person, characterised by an anxious and insecure attachment pattern and absence of impulse control (Castelló, 2005). Difficulties in emotion regulation, an attachment marked by childhood trauma with a style of parental permissiveness, and resentment toward parents show a positive association with substance abuse and emotional dependence (Momeñe et al., 2021b). A predictive effect of emotional dependence on alcohol and drug abuse has been observed, and a mediating effect of emotional dependence on the relationship between childhood trauma and substance addictions has also been found. This implies that, depending on the attachment style received in childhood, emotional dependence would mediate the type of substance abuse (Barbarias et al., 2019).

Likewise, emotional dependence would also be implicated in the existence of non-substance addictions. Adolescence is a vital period characterised by an excess of dependence that can lead to vulnerability to the development of addictions (Estévez et al., 2017). In addition, the current extension and development of technologies have facilitated abuse in the use of electronic devices such as cell phones, Internet, online games, etc. Emotional dependence has been shown to be a predictor of such abuse, and both emotional dependence and abuse have been correlated with symptoms of anxiety, depression and low self-esteem (Estévez et al., 2017).

On the other hand, emotional dependence has been linked to difficulties in emotion regulation, absence of emotional acceptance, limitations in impulse control and interference in goal-directed behaviours (Iruarrizaga et al., 2019). These difficulties have explained the presence of non-substance addictions such as sex addiction, in which a limitation in the use of sexual regulation strategies and difficulties in emotional awareness are observed (Iruarrizaga et al., 2019; Munguía et al., 2021).

A similar phenomenon concerning emotional dependence occurs with eating disorders (EDs). EDs are more prevalent in families in which the development of autonomy is limited, leading to the subsequent search for dependent partner relationships (Momeñe et al., 2020). A greater presence of violent relationships has also been observed in people suffering from an ED and high emotional dependence (Caslini et al., 2016). Risk factors such as impulsivity, fear of maturity, excessive perfectionism and feelings of ineffectiveness are associated with affective dependence on the aggressor partner (Momeñe et al., 2020).

The relationship between emotional dependence and addictive behaviours can be explained by factors such as a deficit in impulse control and an insecure attachment style (Momeñe et al., 2021a). Impulsivity is related to avoidance of isolation and loneliness, the need to please the other, an attachment style based on parental permissiveness, and childhood trauma (Estévez et al., 2018). In fact, regulation of one’s emotions plays a significant role in emotional dependence and psychological abuse in violent relationships. Psychological abuse together with difficulties in emotion regulation have been shown to be predictors of emotional dependence (Momeñe et al., 2017).

Certain personal characteristics such as an inadequate coping style based on self-criticism and isolation, as well as the development of symptoms of depression and anxiety and a profile marked by interpersonal sensitivity and suspicion, influence the relationship between emotional dependence and permanence in violent relationships (Momeñe et al., 2021b). Both young people who have suffered psychological violence and those who have exerted it obtain higher scores in emotional dependence (Estévez et al., 2018; Marcos et al., 2020). Among the factors that have better explained this relationship is self-deception; however, no differences have been found according to sex or age (Martín and de la Villa Moral, 2019). Despite this, men score higher on the need to please, and differentiation is observed between people with and without a partner, who have a greater need for exclusivity and to avoid being alone, respectively (Urbiola et al., 2017).

On the other hand, having addictive disorders and remaining in violent relationships in adolescence do not seem to be isolated facts. High levels of physical violence predict increased substance use in adolescents (Espelage et al., 2018). Similarly, high substance use is associated with increased victimisation by physical and psychological violence (Taylor and Sullivan, 2021).

Different studies relate emotional dependence with addictive behaviours and with permanence in violent relationships. However, no specific studies have been found in the scientific literature that associate these three aspects and provide a global perspective of the effect of emotional dependence on addictive disorders and staying in violent relationships. Precisely, the aim of this study is to analyse the relationship between emotional dependence, addictive behaviours, and remaining in violent relationships in adolescence. Adolescence is a complex period in which many biological and characterial changes happen, which can derive in vulnerability for developing disadaptive behaviour patterns and stablishing first relationships, which can be sometimes toxic (Estévez et al., 2017). The main objective is to determine whether there is a mediating effect of intimate partner violence (IPV) in the relationship between emotional dependence and addictions with or without substances. Considering addictions as a result variable, we aimed to analyse the impact of contextual factors such as received violence, to explore its effects on the apparition of addictive behaviours in people who are characterised by emotional dependence patterns. Received violence could be a determinant and explanatory factor of this relationship between emotional dependence and addictions. We will also explore possible differences in the variables according to the participants’ sex and age.

## Materials and Methods

This study used a non-experimental, cross-sectional correlational design. A probabilistic sampling method was carried out with stratified one-stage random sampling with proportional allocation, and the distribution was based on weight or size within the population. To stratify, the following aspects were considered: the number of educational units, the number of adolescents per class, urban and rural areas, as well as the distribution of grade by sex.

### Participants

Participants in this study had to be adolescents between 14 and 18 years-old and had to have at least one dating relationship for at least 1 month to complete the questionnaire. To calculate the sample size, the confidence level of the sample and the relationship with the margin of error or variation that always exists between the results obtained in a sample and its inference concerning the population were considered. The confidence level was 0.95, with a margin of error criterion of 0.015. Considering the sampling characteristics, a correction factor, estimated at 2, was considered due to the design effect, to expand the sample size and reduce the variability of the observations. Finally, the sample size was increased to compensate for a possible 10% non-response.

### Procedure

The study was carried out after the delivery of informed consent forms to the parents and/or guardians of the adolescents who completed the questionnaires. They were informed about the norms for completion, duration and questions to be answered, the voluntary nature of the study, the confidentiality and anonymity of the data obtained, and contacts of the researchers. During the administration of the questionnaires, a researcher remained in the classroom with the students until all the questionnaires were completed. The teachers remained optionally. The students received a pencil and a certificate of participation as a token of appreciation. This study was carried out following the criteria of the Declaration of Helsinki (World Medical Association, 2013). The protocol received the ethical approval by the Ministry of Education of Portoviejo of the Manabí Province, Republic of Ecuador (No. 13D01-40256).

### Instruments

**Scale of Emotional Dependence** in the courtship of young people and adolescents–DEN (Urbiola et al., 2014). This scale is made up of 12 items structured in 4 subscales: Avoid being alone (α = 0.59), Need for Exclusivity (α = 0.73), Need to Please (α = 0.66), and Asymmetric Relationship (α = 0.59). It is rated on a 6-point Likert-type scale, ranging from 0 (*Never*) to 5 (*Always*). It was an essential requirement to have had at least one dating relationship for at least 1 month to complete the questionnaire. Cronbach’s alpha of 0.82 was obtained in the validation study. For this study, Cronbach’s alpha for the global scale was 0.87.

**The Scale of Violence** Received, Exercised, and Perceived in Dating Relationships of Young People and Adolescents–VREP (Urbiola et al., 2020). This scale is made up of 28 items, integrated into five types of violence: Physical Violence (α = 0.76), Sexual Violence (α = 0.86), Social Psychological Violence (α = 0.77), Psychological Violence Humiliation-Coercion (α = 0.82), and Psychological Violence Control-Jealousy (α = 0.83). It assesses three aspects of violence: exerted, received and perceived. For this specific study, received violence was analysed. The items are rated on a 6-point Likert-type scale: (0 = *Never*, 1 = *Once*, 2 = *From 2 to 5 times*, 3 = *From 6 to 10 times*, 4 = *From 11 to 15 times*, and 5 = *More than 15 times*). Participants had to maintain or have maintained at least one relationship with a partner lasting more than 1 month. They were requested to assess to what extent the situations presented in the questionnaire occurred. In the original study, the scale had a Cronbach’s alpha coefficient of 0.99. In the present study, Cronbach’s alpha for the scale of received violence was 0.94.

**Impulse control disorders**. MULTICAGE CAD 4 (Pedrero et al., 2007). Using 32 items, this instrument assesses eight impulse control disorders and addictions: drug and alcohol use disorder, gambling disorder, substance addictions, EDs, Internet addiction, gaming addiction, compulsive spending and sex addiction. Each scale is evaluated through 4 items that reproduce the CAGE scheme: self-perception of the problem, perception by cohabitants, associated feelings of guilt, and signs of abstinence or inability to control the behaviour. The response format is dichotomous “Yes/No,” in which none or one affirmative response would indicate “no problem,” two affirmative responses indicate the “possible existence of the problem,” three affirmative responses indicate “very probable existence of the problem,” and four affirmative responses indicate “sure existence of the problem.” In the present study, Cronbach’s alpha for the total scale was 0.86.

### Statistical Analysis

First, Student’s *t*-test was used to determine the differences by sex and analysis of variance (ANOVA) by age groups (Group 1 = 14 years; Group 2 = 15 years; Group 3 = 16 years; Group 4 = 17 years; Group 5 = 18 years) in the dimensions of the emotional dependence, IPV, and addictions with or without substances. Scheffe *post hoc* tests were conducted to analyse differences in the means of the established age groups in pairs. Second, the relationship between emotional dependence, received violence, and different types of addictions was analysed through a correlational analysis using Pearson’s *r*.

Third, a mediation analysis was carried out to determine the effect or influence of received violence (as a mediator – M) on the relationship between emotional dependence (as independent variable – IV) and different types of addictions with or without substances (as dependent variable – DV). To perform the statistical analyses, the SPSS programme (Statistical Package for the Social Sciences, 2013) was used. Specifically, for the mediation analysis, the PROCESS programme of SPSS by Hayes and Preacher (2013) was used.

## Results

The sample is made up of 1,533 adolescent students (10th grade of Basic General Education, 1st, 2nd, and 3rd grade of High School) of both sexes, of whom 53.9% are males (*n* = 826) and 46.1% are females (*n* = 707), aged between 14 and 18 years (*M* = 15.76, SD = 1.25) belonging to 12 Fiscal Educational Units of the different urban sociodemographic sectors (63.86%, *n* = 979) and rural (36.13%, *n* = 554) of the Portoviejo Canton of the Manabí Province of the Republic of Ecuador. To obtain the sample, the official organisms of Ecuador (United Nations Commission on Narcotic Drugs, 2005) were considered, a name that is currently known as the Technical Secretariat of Drugs (SETED).

### Differential Profile by Age and Sex

The scores on the variables of interest were compared according to the participants’ sex (see Table 1). The results show that males had a higher level of emotional dependence than females (*M* = 1.40, SD = 1.01, *t* = 9.82, *p* < 0.001, *d* = 0.50). Received violence was also higher in males than in females (*M* = 0.56, SD = 0.67, *t* = 3.72, *p* < 0.001, *d* = 0.19), with statistically significant differences in all subscales except for Psychological Violence based on Control-Jealousy (*t* = 1.54, *p* = 0.124). Statistically significant differences were also observed in all types of addictive behaviours, with higher scores in males in all cases.

**TABLE 1:** Descriptive statistics and mean differences between women and men.

	Females (*n* = 699)		Males (*n* = 820)				
		
	*M*	SD	*M*	SD	*t*	*p*	*d*
DEN_Total	0.93	0.85	1.40	1.01	9.82	<0.001	0.50
DENavoidalone	0.60	0.81	1.08	1.02	10.00	<0.001	0.51
DENexclusive_need	1.36	1.31	1.78	1.36	5.96	<0.001	0.31
DENneed_please	0.85	0.99	1.48	1.25	11.05	<0.001	0.56
DENasymmetric_rela	0.90	1.03	1.25	1.12	6.27	<0.001	0.32
VREPReceived	0.43	0.68	0.56	0.67	3.72	<0.001	0.19
ViolencePhysicalR	0.26	0.61	0.42	0.69	4.75	<0.001	0.24
ViolenceSexualR	0.21	0.61	0.34	0.67	4.02	<0.001	0.20
ViolencePsiSociR	0.39	0.79	0.51	0.77	2.82	<0.001	0.14
ViolencePsiHuR	0.37	0.76	0.53	0.76	4.20	<0.001	0.22
ViolencePsiCelR	0.86	1.09	0.95	1.02	1.54	0.124	0.08
Addictions							
CAGAlcohol	1.06	1.13	1.52	1.27	7.50	<0.001	0.38
CAGGambling	0.79	1.11	1.31	1.25	8.57	<0.001	0.44
CAGDrugs	0.70	1.08	1.23	1.27	8.66	<0.001	0.45
CAGEating D.	1.04	1.29	1.21	1.26	2.60	0.009	0.13
CAGInternet	1.44	1.43	1.68	1.41	3.39	<0.001	0.17
CAGGaming	0.75	1.19	1.46	1.37	10.87	<0.001	0.55
CAGCompul.Spend	0.74	1.03	1.17	0.40	7.53	<0.001	0.57
CAGAdiccSex	0.38	0.86	0.90	1.15	10.13	<0.001	0.51

*n, sample size; M, mean; SD, standard deviation; t, Student’s t; p, significance level. DEN, emotional dependence; DENavoidalone, avoid being alone; DENexclusive_need, Need for Exclusivity; DENneed_please, Need to Please; DENasymmetric_rela, Asymmetric Relationship; VREP Received, Violence Received, Exercised, and Perceive; ViolencePhysicalR, Physical Violence; ViolenceSexualR, Sexual Violence; ViolencePsiSociR, Social Psychological Violence; ViolencePsiHuR, Psychological Violence Humiliation-Coercion; ViolencePsiCelR, Psychological Violence Control. CAG, MULTICAGE CAD 4; CAGAlcohol, alcohol use disorder; CAGGambling, gambling disorder; CAGDrugs, drug use disorder; CAGEating D., eating disorder; CAGInternet, Internet addiction; CAGGaming, gaming addiction; CAGCompul.Spend, compulsive spending; CAGAdiccSex, sex addiction.*

Differences in the variables of interest were analysed according to the age groups (see Table 2). Statistically significant differences were observed for emotional dependence, *F*_(4,1514)_ = 5.51, *p* < 0.001, *d* = 0.15, with higher scores in Groups 3 and 5 (*M* = 1.29, SD = 0.97 and *M* = 1.44, SD = 0.99, respectively). Main significant differences were found between Groups 1 and 3 (*t* = −3.24, *p* = 0.009). There were also statistically significant differences in received violence, *F*_(4,1514)_ = 3.15, *p* = 0.014, *d* = 0.11, with Groups 4 and 5 having the highest scores (*M* = 0.54, SD = 0.74 and *M* = 0.65, SD = 0.79, respectively). Main significant differences in the total received violence were found between Groups 1 and 5 (*t* = −6.83, *p* = 0.034). Regarding addictive behaviours, in general, there were no significant differences between age groups.

**TABLE 2:** Differences in emotional dependence, received violence, and addictive disorders according to age.

	G1 (*n* = 295)		G2 (*n* = 347)		G3 (*n* = 401)		G4 (*n* = 365)		G5 (*n* = 111)			
					
	*M*	SD	*M*	SD	*M*	SD	*M*	SD	*M*	SD	*F*	*p*
DEN	1.01	0.88	1.16	0.98	1.29	0.97	1.15	0.99	1.44	0.99	5.51**	0.001
VREPR	0.40	0.63	0.48	0.63	0.50	0.66	0.54	0.74	0.65	0.79	3.15	0.014
CAGAlcohol	1.23	1.21	1.31	1.21	1.32	1.20	1.31	1.29	1.41	1.21	0.51	0.731
CAGGambling	1.10	1.15	1.05	1.22	1.09	1.21	1.00	1.24	1.19	1.25	0.65	0.627
CAGDrugs	0.97	1.15	0.92	1.19	1.03	1.22	0.98	1.26	1.12	1.33	0.74	0.564
CAGEating D.	1.14	1.31	1.11	1.28	1.18	1.26	1.08	1.27	1.19	1.28	0.41	0.803
CAGInternet	1.67	1.46	1.61	1.45	1.61	1.40	1.46	1.43	1.42	1.30	1.27	0.279
CAGgaming	1.13	1.30	1.11	1.35	1.17	1.31	1.13	1.37	1.15	1.36	0.10	0.982
CAGCompul.Spend	0.91	1.06	0.89	1.13	1.03	1.11	0.98	1.13	1.15	1.19	1.68	0.152
CAGAdiccSex	0.55	0.96	0.63	1.08	0.70	1.06	0.69	1.08	0.86	1.11	2.01	0.091

*n, sample size; M, mean; SD, standard deviation; F, Snedecor F; Age groups, G1, 14 years (Group 1); G2, 15 years (Group 2); G3, 16 years (Group 3); G4, 17 years (Group 4); G5, 18 years (Group 5). DEN, emotional dependence; VREP Received, Violence Received, Exercised, and Perceived; CAG, MULTICAGE CAD 4; CAGAlcohol, alcohol use disorder; CAGGambling, gambling disorder; CAGDrugs, drug use disorder; CAGEating D., eating disorder; CAGInternet, Internet addiction; CAGGaming, gaming addiction; CAGCompul.Spend, compulsive spending; CAGAdiccSex, sex addiction.*

**p ≤ 0.001.

### Relationship Between Emotional Dependence, Received Violence, and Behavioural Addictions

A correlational analysis between the main variables was carried out; specifically, emotional dependence, dimensions of received IPV, and addictive behaviours (see Table 3). Statistically significant correlations were found in all cases. Emotional dependence correlated significantly with received violence (*r* = 0.37, *p* < 0.001). Specifically, the dimension of received violence correlated most strongly with emotional dependence was Psychological Violence Control-Jealousy (*r* = 0.39, *p* < 0.001). Compulsive spending was the addiction most strongly associated with emotional dependence (*r* = 0.21, *p* < 0.001), whereas sex addiction was more strongly associated with received violence (*r* = 0.18, *p* < 0.001).

**TABLE 3:** Analysis of correlations between emotional dependence, addictive behaviours, and received violence.

	1	2	3	4	5	6	7	8	9	10	11	12	13	14	15
1															
2	0.29**														
3	0.24**	0.71**													
4	0.32**	0.65**	0.69**												
5	0.30**	0.72**	0.73**	0.79**											
6	0.39**	0.59**	0.53**	0.71**	0.76**										
7	0.37**	0.82**	0.82**	0.89**	0.93**	0.87**									
8	0.21**	0.12**	0.14**	0.14**	0.12**	0.11**	0.14**								
9	0.19**	0.13**	0.15**	0.14**	0.14**	0.13**	0.16**	0.59**							
10	0.14**	0.09**	0.13**	0.11**	0.10**	0.10**	0.12**	0.53**	0.56**						
11	0.11**	0.07**	0.09**	0.08**	0.10**	0.07**	0.10**	0.40**	0.43**	0.51**					
12	0.17**	0.07**	0.06**	0.08**	0.10**	0.13**	0.11**	0.34**	0.39**	0.38**	0.45**				
13	0.20**	0.11**	0.14**	0.15**	0.15**	0.15**	0.16**	0.41**	0.46**	0.44**	0.37**	0.53**			
14	0.21**	0.13**	0.18**	0.15**	0.14**	0.13**	0.17**	0.43**	0.48**	0.48**	0.46**	0.44**	0.51**		
15	0.16**	0.15**	0.21**	0.13**	0.16**	0.12**	0.18**	0.41**	0.48**	0.50**	0.41**	0.34**	0.46**	0.60**	

*1, DEN_Total, emotional dependence; 2, Physical Violence Received; 3, Sexual Violence Received; 4, Psychological-Social Violence Received; 5, Psychological Violence-Humiliation Received; 6, Psychological Violence-Jealousy Received; 7, Received Violence; 8, CAG Alcohol; 9, CAG Gambling; 10, CAG Drugs; 11, CAG Eating D.; 12, CAG Internet; 13, CAG Gamings; 14, CAG Compulsive spending; 15, CAG Sex Addiction.*

**p ≤ 0.001.

### Mediation Analysis

**FIGURE 1:**
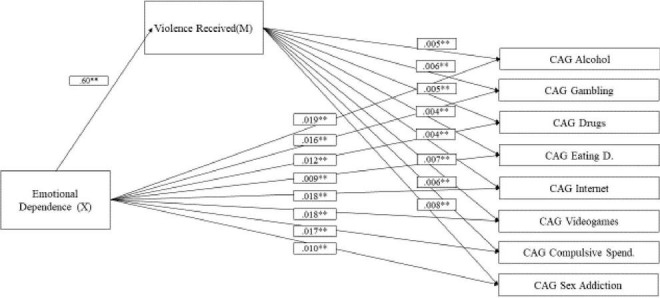
Mediation model of intimate partner violence in the relationship between emotional dependence and addictive behaviours. CAGAlcohol, alcohol use disorder; CAGGambling, gambling disorder; CAGDrugs, drug use disorder; CAGEating D., eating disorder; CAGInternet, Internet addiction; CAGGaming, gaming addiction; CAGCompul.Spend, compulsive spending; CAGAdiccSex, sex addiction. **p ≤ 0.001.

A mediation analysis was conducted to determine whether IPV (M) has a mediating effect on the relationship between emotional dependence (X) and addictive behaviours (Y) (see Table 4). In this case, a mediating effect of received violence on this relationship was observed in all cases (see Figure 1). In other words, received violence exerted mediated in the relationship between emotional dependence and all types of addictive behaviours analysed: alcohol, gambling, drugs, EDs, Internet, video games, compulsive spending, and sex addiction.

**TABLE 4:** Mediation model that includes emotional dependence (X), intimate partner violence (M), and addictive behaviours (Y).

	β	SE	*t*	95% CI
**CAGAlcohol**				
Total effect	0.02	0.01	8.30**	[0.017, 0.027]
Indirect effect	0.02	0.01	6.68**	[0.013, 0.025]
**CAGGambling**				
Total effect	0.02	0.01	7.69**	[0.015, 0.025]
Indirect effect	0.02	0.01	5.78**	[0.011, 0.022]
**CAGDrugs**				
Total effect	0.01	0.01	5.44**	[0.009, 0.020]
Indirect effect	0.01	0.01	4.00**	[0.006, 0.017]
**CAGEating D.**				
Total effect	0.01	0.01	4.29**	[0.007, 0.017]
Indirect effect	0.01	0.01	3.21**	[0.004, 0.016]
**CAGInternet**				
Total effect	0.02	0.01	6.66**	[0.015, 0.027]
Indirect effect	0.02	0.01	5.46**	[0.012, 0.025]
**CAGGaming**				
Total effect	0.02	0.01	7.83**	[0.017, 0.028]
Indirect effect	0.02	0.01	5.89**	[0.012, 0.024]
**CAGCompul.Spend**				
Total effect	0.02	0.01	8.50**	[0.016, 0.025]
Indirect effect	0.02	0.01	6.57**	[0.012, 0.022]
**CAGAdiccSex**				
Total effect	0.01	0.01	6.10**	[0.010, 0.019]
Indirect effect	0.01	0.01	3.87**	[0.005, 0.014]

*CAGAlcohol, alcohol use disorder; CAGGambling, gambling disorder; CAGDrugs, drug use disorder; CAGEating D., eating disorder; CAGInternet, Internet addiction; CAGGaming, gaming addiction; CAGCompul.Spend, compulsive spending; CAGAdiccSex, sex addiction.*

**p ≤ 0.001.

## Discussion

The main objective of this study was to analyse the relationship between IPV, emotional dependence and addictive behaviours. First, sex and age differences in the study participants were explored. Significant sex differences were observed in all variables, with males scoring higher in emotional dependence and received violence, except for psychological violence based on humiliation-coercion. These results contradict previous data showing an absence of significant differences in emotional dependence as a function of sex (Momeñe et al., 2017; Urbiola et al., 2017; Martín and de la Villa Moral, 2019). Males also presented higher scores in all addictions. However, previous studies point to sex differences depending on the type of addiction. For example, being male is associated with greater severity of pathological gambling disorder, or, on the contrary, compulsive spending is higher in females (Estévez et al., 2021). People who are exposed to violent situations may develop higher emotional dependence patterns, which could also contribute to the repetition of suffering or receiving violence patterns in certain situations. A possible explanation would be that males may present a more impulsive pattern (Estévez et al., 2018), which could be explaining higher scores in addictive behaviours and a higher vulnerability to suffer violence, which has been related to the development of emotional dependence (Momeñe et al., 2021b).

Concerning differences by age group, the results revealed significant differences in emotional dependence and received violence, with the highest scores on these scales obtained by those in the oldest age group (18 years). It should be noted that this age group likely has more relationship experience, which could contribute to a higher manifestation of violence situations in the couple. Conversely, no significant differences were found in the age groups in the types of addictions with or without substances, except for addiction to sex, which was also slightly higher in the case of the 18-year-old participants.

Secondly, the relationship between emotional dependence, received violence, and addictions with or without substances was analysed. Statistically significant relationships were found in all cases. Specifically, the type of violence that correlates to a greater degree with the level of emotional dependence is psychological violence based on control and jealous attitudes toward the partner. Previous studies support these results, indicating that emotional dependence has a mediating effect between psychological violence based on control of the partner and factors such as self-esteem (Urbiola et al., 2019). Separation anxiety and fear of loss make it difficult to establish limits in the face of the partner’s attempts of control, blackmail or jealousy. In this type of situation, the affective bond is characterised by being fragile and pathological, which makes separation more difficult in situations of violence (Del Castillo et al., 2015).

Concerning addictive behaviours, compulsive spending stands out as the addiction that correlates the highest with emotional dependence. In this study, sex differences were found in this type of addiction, as males showed higher scores that females in compulsive spending, an information that contradicts data from other studies, showing a greater presence of compulsive spending in females than in males (Estévez et al., 2021). However, it is noteworthy that, in this study, the males obtained higher scores in emotional dependence.

To explore further the influence of IPV on emotional dependence and addictions, a mediation analysis was conducted. The data obtained showed a mediating effect of IPV on the relationship between emotional dependence and all substance and non-substance addictions (alcohol, gambling, drugs, EDs, Internet addiction, video game addiction, compulsive spending, and sex addiction). In all cases, received violence had a significant influence on the relationship. It has been found that high levels of emotional dependence together with experiences of IPV are strongly associated with the development of addictive behaviours. Previous studies confirm the relationship between emotional dependence and victimisation in young people’s intimate partner relationships (Martín and de la Villa Moral, 2019).

Likewise, the existence of traumatic experiences in childhood helps to explain the presence of addictive disorders. In the specific case of EDs, it is observed that difficulties in emotion regulation preceded by situations of maltreatment in childhood can predispose individuals to the development of EDs (Huston et al., 2019; Groth et al., 2020; Monteleone et al., 2020). Experiences of childhood maltreatment and a particular attachment style can, in turn, explain the existence of emotional dependence and victimisation in young people’s dating relationships (Barroso, 2014; Estévez et al., 2018; Momeñe and Estévez, 2018).

Other addictions such as pathological gambling, Internet abuse, and video game addiction present a similar aetiology (Estévez et al., 2014). Inadequate emotion regulation and high emotional dependence associated with early childhood trauma experiences may precede the development of addictive behaviours. Compulsive sexual behaviour can also be explained by these types of factors (Iruarrizaga et al., 2019).

As can be observed, emotional dependence is related to impulsivity, which is associated with avoidance of being alone, asymmetrical relationships, a need to please, and, in turn, with childhood trauma, often consisting of physical and/or psychological abuse (Estévez et al., 2018). A traumatic history of abuse linked to an impulsive personality and preceded by difficulties in emotion regulation and dependence could lead to the development of addictive behaviours (Jiménez-Murcia et al., 2015). In this sense, the importance of resilience or seeking social support as protective factors stands out. The presence of resilient resources contributes to the decrease in the levels of emotional dependence and psychological abuse (López-Fuentes and Calvete, 2015; Momeñe and Estévez, 2019).

This study is not without limitations. Firstly, it is a cross-sectional study, which prevents generalisation of the results obtained. Future research could extend the study by carrying out successive evaluations so that the predictive effects of the variables could be analysed. Secondly, there is a limitation concerning the age of the participants in the sample, which is limited to 14–18 years of age. It would be advisable to extend the study of the variables analysed to other periods of life to explore possible differences. Furthermore, the requirement of having to had at least one relationship last for a month could be limited considering that may have not been much time for manifesting problematic relationship behaviours. Finally, it would be interesting to investigate in greater depth the specific emotion regulation strategies of people with high emotional dependence and the presence of addictive behaviours who have been exposed to situations of IPV. This information would help to discriminate strategies that promote adequate coping with the situation from strategies that perpetuate abuse and addictive impulses, which make it difficult to implement adequate psychological resources (Sancho et al., 2019).

In conclusion, the results of this study confirm the hypothesis that IPV has a mediating effect on the relationship between emotional dependence and the existence of addictive behaviours with or without substance. People characterised by high emotional dependence present significant affective needs that prevent the establishment of limits and are associated with addictions such as pathological gambling, sex addiction, compulsive spending, etc. In this sense, the experience of physical and/or psychological abuse within the couple is a risk factor for the perpetuation of addictive behaviours. Psychotherapeutic work could help to reduce the impact of emotional dependence in dating relationships and also reducing impulsive patterns that could prevent adolescents from developing addictive behaviours. For this purpose, we should promote affective education, as well as the design of therapies based on coping with violent situations, which would allow the development and increase of resilient personal resources and adequate coping strategies.

## Data Availability Statement

The raw data supporting the conclusions of this article will be made available by the authors, without undue reservation.

## Ethics Statement

The studies involving human participants were reviewed and approved by the District of Portoviejo Canton of the Manabí (Ministry of Education, Province of the Republic of Ecuador). Written informed consent to participate in this study was provided by the participants’ legal guardian/next of kin.

## Author Contributions

II, LO, and MDC contributed to conception and design of the study. II and AE organised the database. AE, PM, and JM performed the statistical analysis. PM and JM wrote the first draft and different sections of the manuscript. All authors contributed to manuscript revision, read, and approved the submitted version.

## Conflict of Interest

The authors declare that the research was conducted in the absence of any commercial or financial relationships that could be construed as a potential conflict of interest.

## Publisher’s Note

All claims expressed in this article are solely those of the authors and do not necessarily represent those of their affiliated organizations, or those of the publisher, the editors and the reviewers. Any product that may be evaluated in this article, or claim that may be made by its manufacturer, is not guaranteed or endorsed by the publisher.
